# Coronary calcium scoring: are the results comparable to computed tomography coronary angiography for screening coronary artery disease in a South Asian population?

**DOI:** 10.1186/1756-0500-6-279

**Published:** 2013-07-19

**Authors:** Nizar Bhulani, Ali Khawaja, Asif Jafferani, Maryam Baqir, Ramin Ebrahimi, Zafar Sajjad

**Affiliations:** 1 Department of Medicine, Research Associate, Aga Khan University, Karachi, Pakistan; 2 Medical College, Aga Khan University, Karachi, Pakistan; 3 Department of Biological and Biomedical, Sciences, Teaching Assistant, Aga Khan University, Karachi, Pakistan; 4 Department of Medicine, Division of Cardiology, University of California Los Angeles, Los Angeles, California, USA; 5 Department of Radiology, Associate Professor and Chair, Aga Khan University Hospital, Karachi, Pakistan

**Keywords:** Coronary calcium scoring, CT Coronary artery, Coronary artery disease, Stenosis, Pakistan, South Asia

## Abstract

**Background:**

The need of having feasible screening tools like Coronary Calcium Scoring (CCS) and CT Coronary Artery (CTCA) for Coronary Artery Disease (CAD) has become paramount. We aimed to evaluate the accuracy of CCS in determining the degree of stenosis of coronary vessels as compared to that determined by CTCA in a South Asian population.

**Methods:**

A retrospective study was conducted at The Aga Khan University Hospital. A total of 539 patient records were reviewed who had undergone CCS and CTCA between 2008 and 2010. Patient records were reviewed by comparing their CCS and CTCA results.

**Results:**

About 268 out of 301 (89%) patients with a CCS of 0–9 were found to be free of stenosis on CTCA. On a CCS of 10–99, 110 out of 121 (91%) patients were either free of stenosis or had mild stenosis. About 66 out of 79 (84%) patients had moderate or severe stenosis with a calcium score of 100–400 while none of the patients were free of stenosis. Around 28 out of 38 (74%) patients with a CCS of more than 400 had severe stenosis. However, only 04 patients (11%) were found to have mild stenosis. Spearman’s rho revealed a correlation coefficient of 0.791 with a p-value of <0.001.

**Conclusion:**

Our study reaffirms that in South Asian population, low CCS (<100) is associated with no or minimal stenosis while high CCS warrants further investigation; hence, making it a reliable tool for screening patients with CAD.

## Background

South Asian population is regarded as one of the most susceptible populations to coronary artery disease (CAD) [1]. Already an important cause of morbidity and mortality in developed countries, CAD has started taking its toll on developing countries as well. Mohan et al. reported an overall prevalence of 11% of CAD in a native urban South Indian population whereas Jafar et al. reported around 27% people to be suffering from CAD in Karachi, Pakistan [2,3]. This necessitates the development of better techniques for evaluation of the disease that are feasible with minimal invasiveness and side effects, in order to facilitate early interventions.

Recent progress in imaging techniques has given us the liberty to assess the development and progression of CAD. The advent of computed tomography coronary angiography (CTCA) has increased our knowledge regarding the pathophysiology of CAD [4]. It is a beneficial, non-invasive technique that promises a high degree of accuracy in ruling out CAD. With high feasibility and little procedural risk, CTCA represents a reliable and swift method of directly identifying the extent and severity of coronary artery stenosis [5,6]. Visualization and evaluation of the coronary artery anatomy, morphology and lesions have been made more convenient by this technique with the help of a higher temporal and spatial resolution [4]. Though it may not provide the degree of image quality and diagnostic accuracy compared to invasive coronary angiography, its sensitivity and specificity of 97.4% and 97.8%, respectively with positive predictive value and negative predictive value of 92.2% and 100%, respectively along with a diagnostic accuracy of 96.5% is impressive [7].

Another imaging modality, coronary calcium scoring (CCS) may serve as a strong indicator of cardiac pathological processes such as atherosclerosis which can be measured either by fast electron beam computed tomography (EBCT) or multi-detector computerized tomography (MDCT) [8]. It provides an indication of coronary plaque load and allows risk stratification in patients [9]. Such has been its impact that it has been regarded to be more sensitive and specific in estimating the risk of coronary events than the conventional Framingham risk score [10]. Moreover, a study found a progressive increase in the adjusted risk score of coronary events with simultaneous increase in calcium score in four racial groups [11].

CCS is a relatively cheaper modality with decreased exposure to radiation as compared to CTCA. Hence, it could be used as a more feasible screening tool in patients with CAD. However, data comparing the extent to which the results of CTCA and CCS complement each other remain scarce in the South Asian population. We aimed to evaluate the accuracy of CCS in determining the degree of stenosis of coronary vessels as compared to that determined by CTCA in a South Asian population.

## Methods

The study was carried out at the Department of Radiology, The Aga Khan University Hospital (AKUH), Karachi. AKUH is a major tertiary care hospital serving more than 10 million people of Karachi and the surrounding region. With an operational strength of 650 beds, the facility serves over 42,000 inpatients and over 500,000 outpatients annually. Established since 1985, it is one of the few teaching hospitals in South Asia accredited by the Joint Commission for International Accreditation [12]. A retrospective study design was conducted in which the medical record numbers of patients who had undergone CTCA and CCS were obtained.

### Inclusion and exclusion criteria

All the patients undergoing both CTCA and CCS between February 2008 and February 2010 were included in the study. No patients were excluded.

Scans for the procedures had been acquired on a Toshiba Aquilion 64-row detector computerized tomography scanner (Toshiba Medical Systems, Japan) using cardiac gating. All the scans were read by two professional radiologists separately and the final result was documented. The procedures were carried out as below:

### Coronary calcium score

After obtaining informed consent, a non contrast scan extending from the pulmonary hilum to the base of the heart was obtained in a single breath hold. Volume data was reconstructed in 3 mm axial images. This data was then transferred to a Vitrea 2 Workstation (Vital Images, USA). The coronary artery calcium was manually tagged. The calcium score was calculated using the Agaston system of scoring [13].

### Computed tomography coronary angiography

The indications for CTCA included presence of chest pain and risk factors for CAD, positive (or equivocal) stress test, preoperative assessment prior to valve surgery, evaluation for cardiomyopathy and screening for CAD.

After obtaining informed consent, the patients’ renal function were pre-assessed by serum creatinine levels and they were asked to abstain from caffeine, tobacco and other stimulants for 24 hours prior to the scan. The patients were pre-treated with an oral beta-blocker; Metoprolol 50 mg 12 hours and 2 hours prior to the scan while calcium channel blockers were substituted if there were any standard beta-blocker contraindications to target an optimal heart rate for the scans in the range of 55–65 beats per minute. Intra venous access was obtained in the ante-cubital fossa with a 20G cannula. 80 mLs of non-ionic contrast containing 350 mg of Iodine/mL was injected at a rate of 4 mLs/sec followed by 40 mLs of normal saline. Scan was obtained in single breath hold from the pulmonary hilum to the base of the heart. The data was retrospectively gated and reconstructed at 70, 80 and 30% of the RR interval. Further reconstructions were carried out as required to assess individual segments. Data was transferred to a Vitrea 2 workstation (Vital Images USA) for analysis.

Age, gender, degree of coronary stenosis as obtained through the CTCA and calcium score of the subject as obtained from the CCS were acquired from the records. The classification of degree of stenosis was as follows; mild <30%, moderate 30-60% and severe > 60%. Risk factors for developing CAD like smoking, fasting lipid levels, family history of CAD etc. were not evaluated. The data was entered and analyzed using Statistical package for social science (SPSS 19.0 copyright © SPSS; 1989–02). Descriptive analysis was carried out for the data. The degree of stenosis was divided into four groups namely i) free of stenosis ii) mild iii) moderate and iv) severe, whereas calcium scores were divided into ranges of i) 0–9 ii) 10–99 iii) 100–400 and iv) > 400. As the data was non parametric, Spearman’s rho was performed to assess the correlation between the degree of stenosis and calcium scores.

### Ethical approval

Due to the retrospective nature of the study, a written informed consent could not be obtained from the participants. However, identification of the study participants was kept strictly confidential and approval was taken by the Ethics Review Committee of the Aga Khan University Hospital prior to commencement of the study.

## Results

A total of 539 patients were reviewed retrospectively. The mean age was 50.8 ± 10.3 years, 389 (72%) patients were males (Table 1).

**Table 1:** Frequency analysis of patients undergoing computed tomography coronary angiography and coronary calcium scoring

**Degree of stenosis on computed tomography coronary angiography**	**Number of patients (%)**
**Free of stenosis**	289 (54)
**Mild**	116 (21)
**Moderate**	65 (12)
**Severe**	69 (13)
**Coronary Calcium Score**	
**0-9**	301 (56)
**10-99**	121 (22)
**100-400**	79 (15)
**>400**	38 (7)

### Calcium score at different levels of the degree of stenosis

It was found that 268 (89%) out of 301 patients with a calcium score of 0–9 were free of stenosis on CTCA while only 8% had moderate to severe stenosis.

In patients with a calcium score of 10–99, majority (91%) were either free of stenosis or had mild stenosis. The remaining patients had either moderate or severe stenosis.

In patients with calcium score between 100–400, more than half of the patients; 55% had moderate stenosis while none of the patients were free of stenosis. However, about 29% of the patients were affected with severe stenosis.

In patients with a calcium score of > 400, approximately three quarters of them (74%) had severe stenosis. However, only 11% of patients were found to have mild stenosis. Table 2 summarizes the frequency analysis between calcium scores and degree of stenosis. Spearman’s rho revealed a correlation coefficient of 0.791 with a p-value of < 0.001.

**Table 2:** Frequency analysis showing the comparison between the degree of stenosis and groups of coronary calcium scoring

**Calcium score**	**Degree of stenosis**	**Number of patients (%)**
0-9	Free of stenosis	268(89.0)
	Mild	10(3.3)
	Moderate	7(2.3)
	Severe	16(5.3)
	Total	301(100)
10-99	Free of stenosis	21(17.4)
	Mild	89(73.6)
	Moderate	9(7.4)
	Severe	2(1.7)
	Total	121(100)
100-400	Mild	13(16.5)
	Moderate	43(54.4)
	Severe	23(29.1)
	Total	79(100)
>400	Mild	4(10.5)
	Moderate	6(15.8)
	Severe	28(73.7)
	Total	38(100)

## Discussion

Advances in the imaging modalities in the last decades have raised the expectations of patients to have access to simpler and non-invasive techniques to reach a diagnosis. CTCA is being increasingly used as both, a screening and a diagnostic modality whereas CCS is mainly used for the purpose of risk stratification. CCS is highly cost effective and if accurate in predicting the degree of stenosis, may reduce the number of patients referred for diagnostic procedures lowering the cost burden. Hence, there was a need to compare CTCA and CCS as screening modalities.

CTCA is a non-invasive radiological procedure that allows obtaining quick images with a higher temporal and spatial resolution. It allows direct visualization and evaluation of the coronary artery anatomy, morphology and any lesions within [4]. Moreover, a high degree of accuracy has been validated in identifying the extent and severity of coronary artery stenosis [7]. CCS, on the other hand is an established marker for atherosclerosis and a predictor of cardiac events. It is used to locate and quantify coronary artery calcification [9,14]. A higher score predicts higher plaque burden and a greater chance of cardiac events. CCS is better suited to identify patients with disease rather than to rule out disease or to localize it to particular arterial segments [15] while diagnostically, CTCA’s supremacy has been proven in ruling out disease where CCS has been proven to be inadequate [16,17]. CTCA, however, is a modality which is not free of risks. High doses of X-ray radiations particularly in women and young patients are a serious concern which could lead to serious long-term effects [18]. Keeping the adverse effects in mind, it has been reported that due to low probability in finding non-calcified plaques in the subgroup of negative CCS, CTCA might not always be justified [19]. Keeping this in mind, modalities with fewer side effects like CCS must be brought into practice and justified to screen for CAD; which paved way for this study. The main outcome of this study suggests that increasing CCS correlates with the severity of CAD. However, it also confirms that the extremes of CCS do not necessarily rule out or rule in severe CAD.

In the present study, CTCA showed 89% of the patients to be free of stenosis with a CCS of 0–9. This is consistent with the studies carried out by Werkhoven et. al and Lau et. al who reported 80% and 84% of the patients having no stenosis with a CCS of 0 respectively [15,20]. However, around 5% of our patients in this sub group had severe stenosis. Werkhoven et.al observed significant CAD in 4% of patients with a CCS of 0 compared to 8% detected by Cademartiri et al. and Akram et al. [16,20,21]. Presence of stenosis in the absence of CCS is due to early atherosclerosis which contains lipid-rich plaques which have not calcified. These plaques are proven to be more susceptible to rupture as compared to relatively stable calcium plaques [22,23] making it essential to diagnose and locate them as early as possible. Having said that, histological analysis remains superior to all other modalities [15]. However, its utility in clinical practice remains limited.

In the present study, CCS between 10 and 99 was highly associated with either no or mild stenosis. The intermediate CCSs, on the other hand were less conclusive. Although 54% of the patients with 100–400 CCS had moderate stenosis, 29% had severe disease. Leber, in one of his reviews, mentioned that scores that fall in the intermediate percentiles are not statistically significant [24]. On the other extreme of CCS of > 400; however, the percentage of severe stenosis was the highest at 73.7%. This association, between a high CCS and increased severity in stenosis, is corresponding to the literature which suggests that there is a significantly higher chance of cardiac events in this subgroup of patients [11,15,19,25]. Lau et. al reported that a CCS greater than or equal to 400 increased the sensitivity of CTCA from 93% to 100% [15]. Hence, it may help diagnose disease that is not detected at CT angiography.

A factor which cannot be ignored particularly in the developing countries like Pakistan, is cost effectiveness and patient’s feasibility, hence the need of better application of CCS as a screening modality if CTCA could not be afforded by the patient.

### Limitations

Some limitations of the study need to be addressed. Firstly, there were an unequal number of patients in different groups stratified on the basis of CCS. However, this difference was inherent in this study being a retrospective analysis of the hospital data. Secondly, our study did not take into account the demographics and other risk factors which define the cardiovascular profile of the patients. This also was not that relevant to the study question and; therefore, a more comprehensive study can be designed which can include the above with the screening modalities. This would enable a risk score based on these risk factors along with CCS to be generated for the South Asian population on the lines of the Framingham risk score, which would further help in risk stratification of patients with suspected CAD. Thirdly, this study was not designed to calculate the sensitivity, specificity and positive and negative predictive values for CCS. Lastly, we compared CCS as a screening modality to CTCA and not the invasive coronary angiography which is considered the gold standard for diagnosing CAD.

## Conclusion

The complementary use of CTCA and CCS provides an optimal diagnosis in stenosis detection. A rise in CCS levels simultaneously raises the probability of significant CAD and presents a good and feasible alternative screening modality. It is also emphasized that absence of CCS does not necessarily rule out a disease and vice versa. Our study reaffirms in the South Asian population that low CCS (<100 in our study) is highly indicative of no or minimal stenosis. In this subgroup, further workup should only be carried out in high risk patients. On the other hand, a high CCS in most cases warrants further investigations such as CTCA or invasive coronary angiography to reach a confirmatory diagnosis.

## Competing interest

The authors have no financial or other competing interests to declare.

## Authors’ contributions

NB: Conception, design and drafting of manuscript. AK: Drafting of manuscript and data collection. AJ: Analysis, Interpretation and acquisition of data. MB: Analysis and interpretation of data. RE: Critical Revision and Gave Final Approval. ZS: Conception, Critical review, added intellectual content. All authors read and approved the final manuscript.
